# Electron Spin Selective Iridium Electrocatalysts for the Oxygen Evolution Reaction

**DOI:** 10.1021/acsmaterialsau.3c00084

**Published:** 2023-11-29

**Authors:** Carlos J. Mingoes, Bob C. Schroeder, Ana B. Jorge Sobrido

**Affiliations:** † School of Engineering and Materials Science, Queen Mary University of London, Mile End Road, London E1 4NS, U.K.; ‡ Chemistry Department, University College London, 20 Gordon Street, London WC1H 0AJ, U.K.

**Keywords:** chirality, chiral-induced spin selectivity (CISS), electrocatalysts, oxygen evolution reaction, iridium nanoparticles, electron spin selectivity

## Abstract

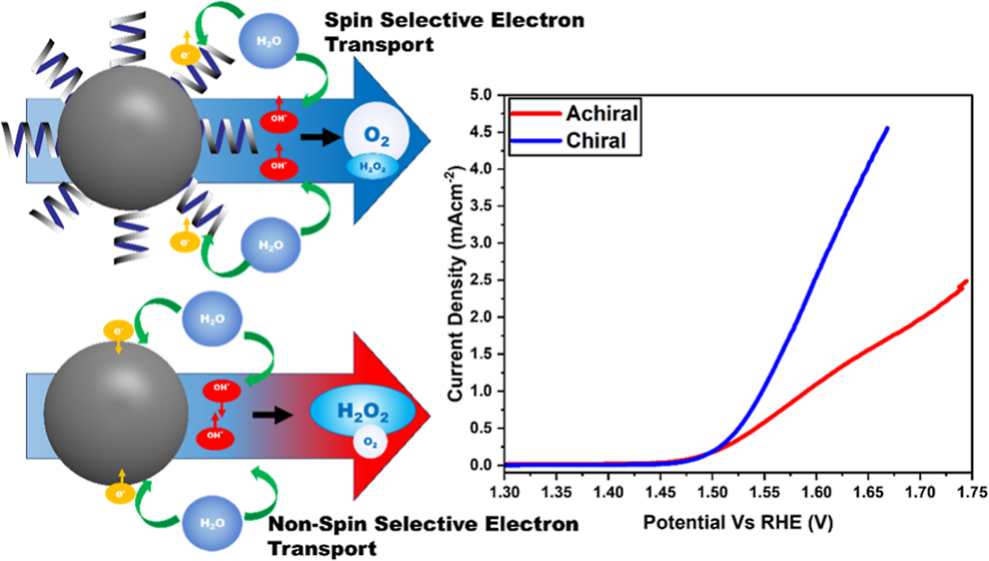

Highly efficient electrocatalysts for water electrolysis are crucial to the widespread commercialization of the technology and an important step forward toward a sustainable energy future. In this study, an alternative method for boosting the electrocatalytic activity toward the oxygen evolution reaction (OER) of a well-known electrocatalyst (iridium) is presented. Iridium nanoparticles (2.1 ± 0.2 nm in diameter) functionalized with chiral molecules were found to markedly enhance the activity of the OER when compared to unfunctionalized and achiral functionalized iridium nanoparticles. At a potential of 1.55 V vs Reference Hydrogen Electrode (RHE), chiral functionalized iridium nanoparticles exhibited an average 85% enhancement in activity with respect to unfunctionalized iridium nanoparticles compared to an average 13% enhancement for the achiral functionalized iridium nanoparticle. This activity enhancement is attributed to a spin-selective electron transfer mechanism taking place on the chiral functionalized catalysts, a characteristic induced by the chirality of the ligand. This alternative path for the OER drastically reduces the production of hydrogen peroxide, which was confirmed via a colorimetric method.

## Introduction

With the ever-increasing energy usage and demand in today’s highly technological society, the need for alternative, sustainable large-scale clean energy production has never been greater.^1^ This has effectively boosted the drive for research into technologies that are geared toward limiting, eliminating, and potentially reversing the adverse environmental impacts generally associated with globalized energy production.^2−4^

Water electrolysis is a very important process under constant development in striving for a sustainable future.^5−10^ This advancement entails the research and production of efficient electrocatalytic materials, capable of offsetting the sluggish kinetics associated with the complex reaction pathways of the oxygen evolution reaction (OER).^9,11−14^ To this avail, a wide range of catalytic nanomaterials of noble and non-noble metal-based, carbides, sulfides, and carbon composites have all been made to tackle this issue.^15−21^

Many of the strategies to design these electrocatalysts are in line with conventional approaches to enhance catalytic activity; optimizing the binding energy efficiencies of the electrocatalyst surfaces toward reactive intermediate species and increasing electrocatalytic surface area. Most recently, unconventional approaches such as inducing spin-selective electron transfer mechanisms and applying external magnetic control to the electrocatalytic systems have been demonstrated to improve the production of OER.^22−25^

Reported research has explored in detail the spin-selective transport of electrons through chiral molecules, that is, there is a spin polarization/imbalance between spin-up and spin-down-orientated electrons.^26−30^ This phenomenon is called the chiral-induced spin selectivity (CISS) effect and it is reported to be applicable in inducing spin-selective electron transfer in electrocatalysis processes such as OER.^31^ Naaman and co-workers have led and developed extensive research into this phenomenon and its applicability in electrocatalysis.^22,26,29,32−44^

By selectively controlling the transfer of electrons based on their spin orientation, the overpotential required to split water into oxygen and hydrogen can be decreased through selective control of the reaction intermediates formed during the OER process, effectively forcing the formation of more favorable intermediate species that will undergo reactions on a lower potential energy surface.^22,41,43^ During water splitting, the interaction of OH radicals intermediates is key in the formation of oxygen, resulting in either the generation of O_2_ the desired product, or H_2_O_2_ a side and parasitic product. The electron spin orientations of the unpaired electron among the OH intermediates produced during the OER are random, giving the reaction an added degree of freedom in terms of the reactions they can undergo. By forcing OH radical intermediates to maintain the same electron spin orientation of their unpaired electron, this degree of freedom can be eliminated, effectively maximizing the efficiency in the OER. It is suggested that forcing oxygen evolution to take place on the ground state triplet oxygen potential energy surface is more efficient and can lead to lower overpotentials.^43^ This reaction path should also significantly reduce the level of formation of hydrogen peroxide (H_2_O_2_).

In this study, we demonstrate the use of chiral molecular functionalization on a state-of-the-art electrocatalytic material (Ir nanoparticles) to enhance its activity toward the OER. This serves as a basis for the potential use of chiral modifications of electrocatalysts.

## Results and Discussion

### Characterization of Iridium Nanoparticles

Iridium nanoparticles were synthesized and further functionalized by adopting and modifying a procedure previously reported, where Ir(III) chloride is thermally reduced to Ir metal nanoparticles by refluxed heating in propanediol before being subsequently functionalized after stirring within a solution containing the ligands overnight (full details on synthesis can be found in the Experimental Section).^45,46^ Nanoparticles functionalized with cysteamine, mercaptopropionic acid, l-cysteine, and d-cysteine are denoted as IrNp@CyA, IrNp@MPA, IrNp@LCy, and IrNp@DCy, respectively, while bare iridium nanoparticles are denoted IrNp. Table 1 shows the list of ligands used, with their molecular structures and chiral orientation.

**Table 1:**
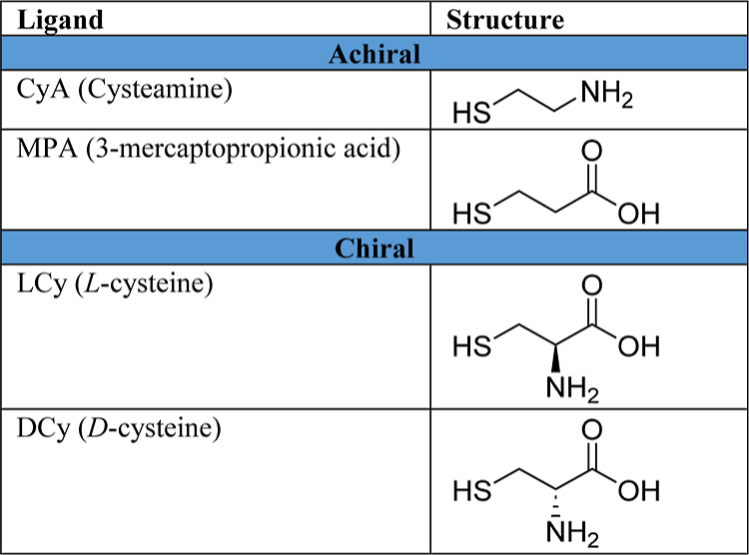
List of Ligands Used to Passivate Iridium Nanoparticles

**Figure 1:**
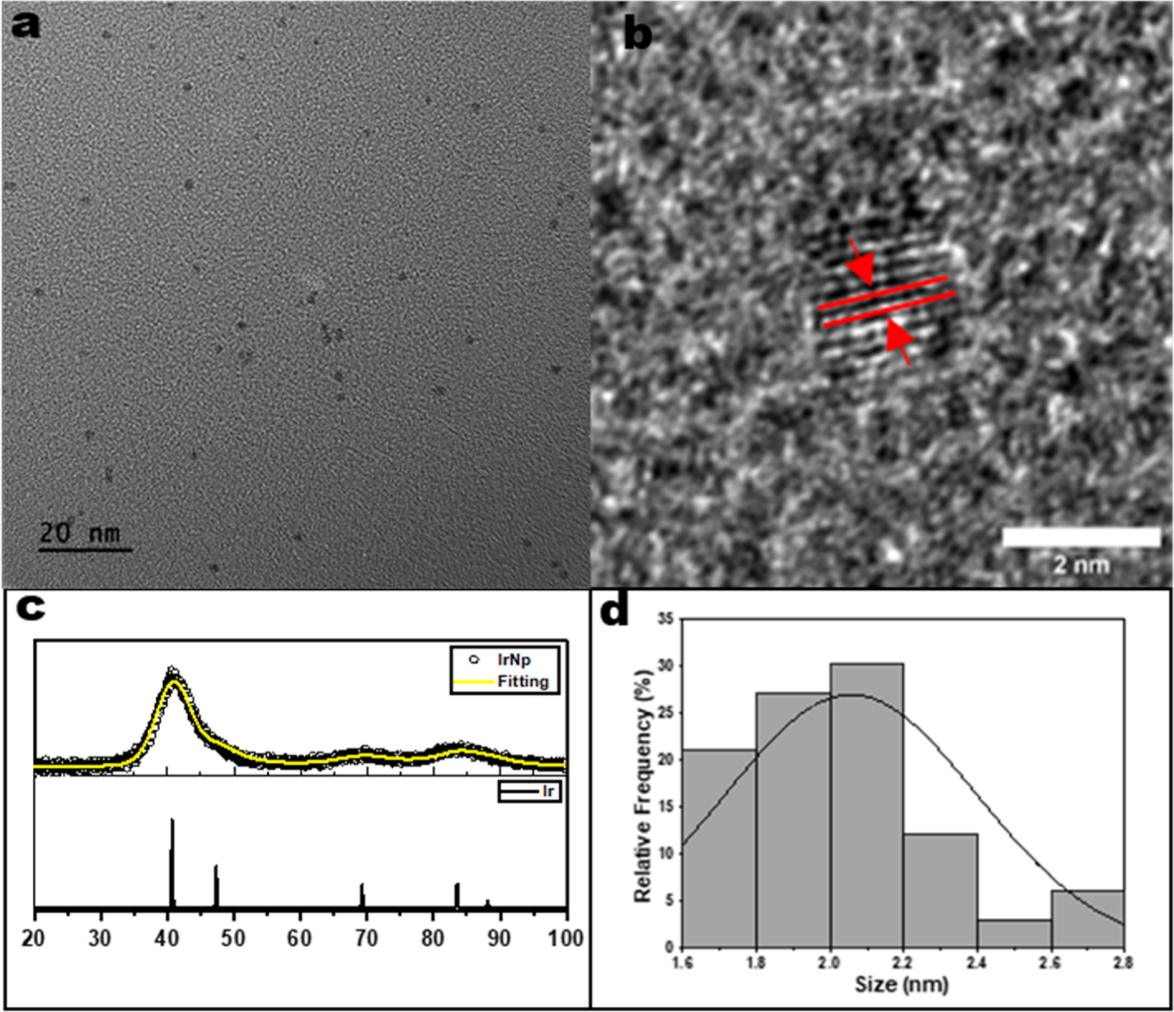
(a,b) TEM images of iridium nanoparticles, (c) X-ray diffraction (XRD) spectrum of iridium nanoparticles, and the (d) corresponding core size distribution.

Transmission electron microscopy (TEM) revealed nanoparticles with a spherical morphology (Figure 1), with an average diameter of 2.1 ± 0.2 nm, and diameters ranging from 1.4 to 2.8 nm (Figure 1d). High-resolution images of these nanoparticles show defined lattice fringes with interplanar spacings equivalent to 0.22 nm.^47^ This interplanar spacing is consistent with the interplanar spacings observed for the pure Ir(111) facets. Similar characteristics are observed for functionalized nanoparticles highlighting no morphological changes to the nanoparticles after undergoing passivation (Figure S2).

XRD data (Figure 1c) support these results. The XRD pattern displayed broad peaks typically observed for nanoparticulated materials. The peaks appeared at 2θ values of 40.8, 47.5, 69.5, 83.9, and 88.5 corresponding to the [111], [200], [220], [311], and [222] Ir metal crystallographic planes. Ir standard obtained is also plotted in Figure 1c as a comparison.^48^ This crystal structure implies the complete reduction of Ir(3+) in the salt to Ir(0) during nanoparticle formation.^49^ Similar XRD spectra (Figure S3) were obtained for the functionalized nanoparticles, boasting similar peak positions and implying no major changes to the crystal structure of the nanoparticles following functionalization. The complete XRD data and TEM images are provided in the Supporting Information (Figures S2 and S3).

**Figure 2:**
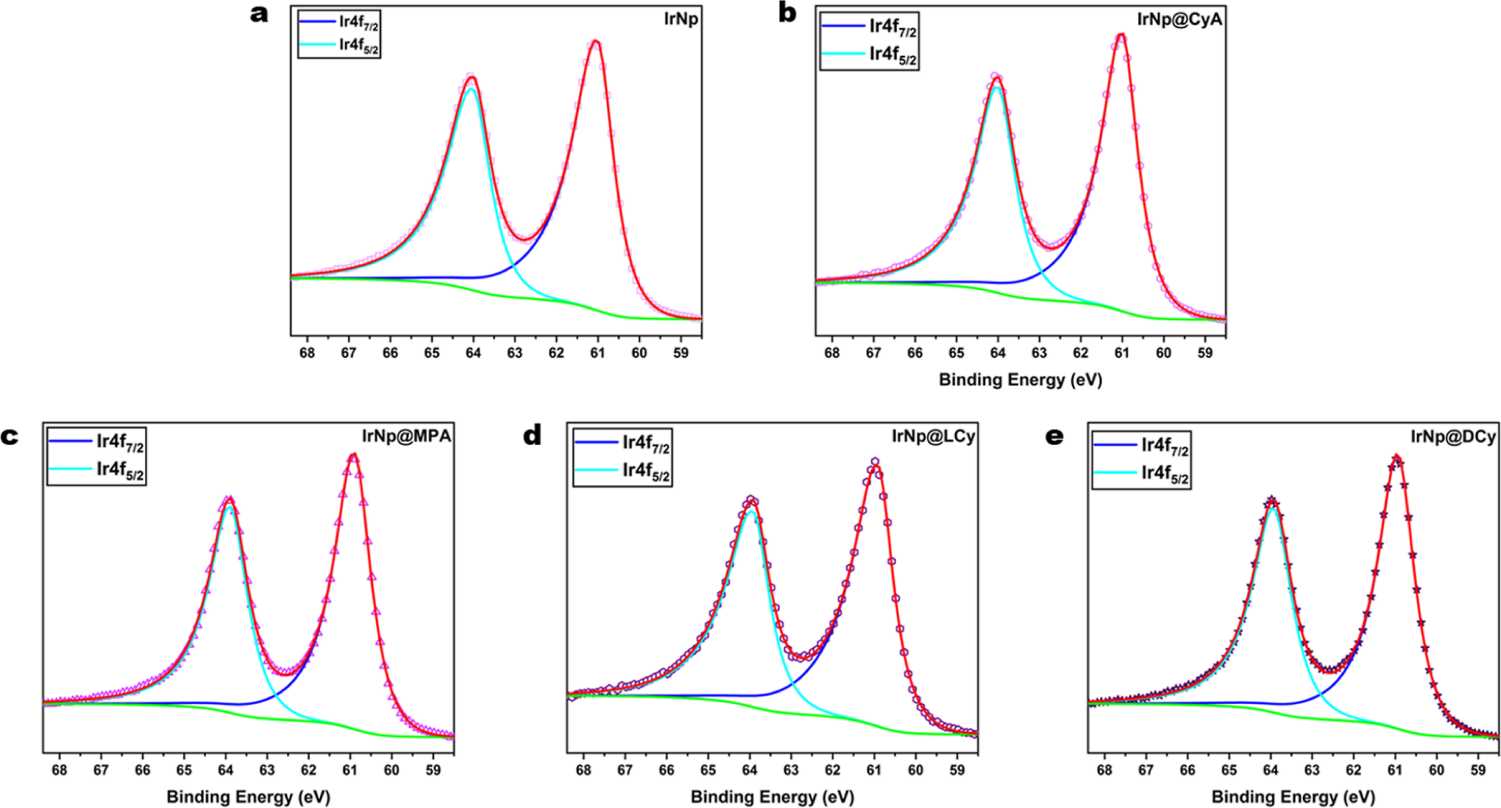
High-resolution Ir 4f XPS spectra for, (a) IrNp, (b) IrNp@CyA, (c) IrNp@MPA, (d) IrNp@LCy, and (e) IrNp@DCy; unfunctionalized, cysteamine, mercaptopropionic acid, and l and d-cysteine functionalized iridium nanoparticles, respectively.

X-ray photoelectron spectroscopy (XPS) characterization of the nanoparticles was carried out to verify the surface functionalization with the various ligands as well as further confirm the oxidation state of the core iridium nanoparticles. High-resolution Ir 4f spectra for all nanoparticles are shown in Figure 2. Ir 4f spectra were fitted with doublets having spin–orbit couplings of 3.0 eV. As shown in Figure 2, all samples were fitted with an Ir 4f (4f_7/2_/4f_5/2_) doublet representative of the pure Ir metal. The respective Ir metal peak binding energies obtained for IrNp, IrNp@CyA, IrNp@MPA, IrNp@LCy, and IrNp@DCy were 61.02, 61.01, 60.91, 60.92, and 60.94 eV, respectively.^50−52^ All binding energies are consistent with an iridium core across all samples, with only small differences not large enough to draw any significant meaning.

**Figure 3:**
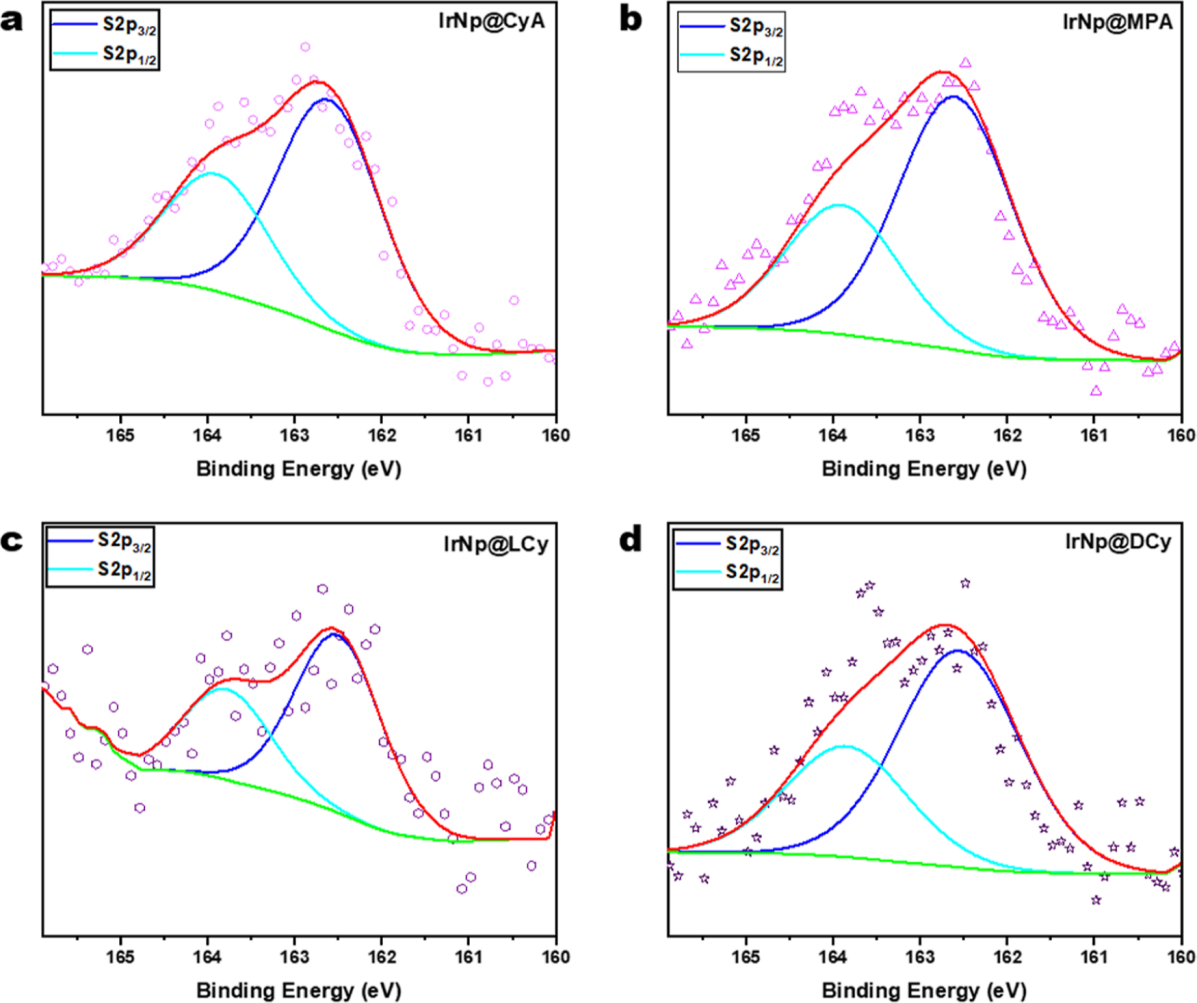
High-resolution S 2p XPS spectra for (a) IrNp@CyA, (b) IrNp@MPA, (c) IrNp@LCy, and (d) IrNp@DCy; cysteamine, mercaptopropionic acid, and l and d-cysteine functionalized iridium nanoparticles, respectively.

High-resolution S 2p (2p_3/2_/2p_1/2_) spectra for all samples were fitted with doublets having spin–orbit couplings of 1.2 eV. These fittings are plotted in Figure 3. The peak binding energies for IrNp@CyA, IrNp@MPA, IrNp@LCy, and IrNp@DCy were 162.60, 162.60, 162.50, and 162.55 eV, respectively. These binding energies are consistent with metal-bounded thiol, supporting the successful functionalization of iridium nanoparticles.^53,54^

**Figure 4:**
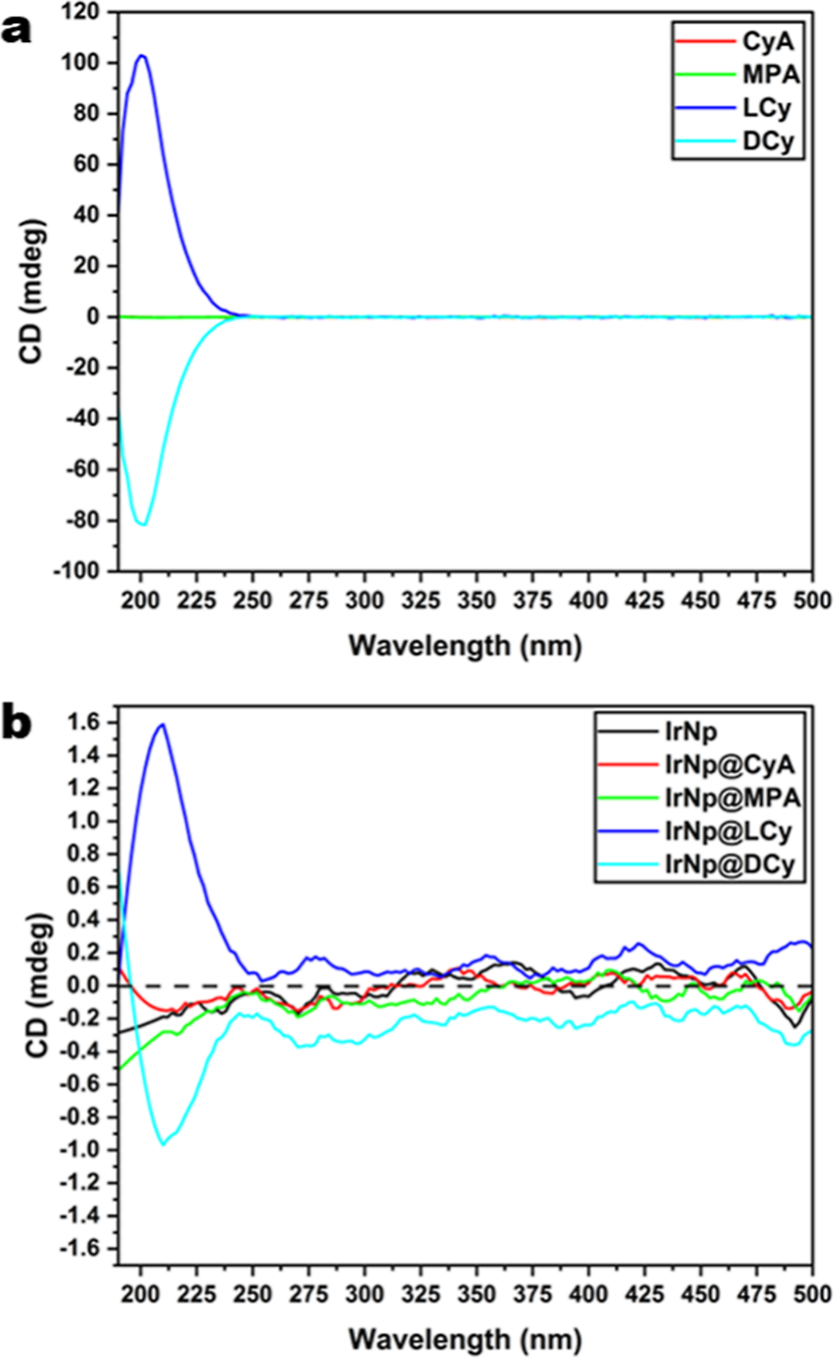
(a) CD spectra for ligands and (b) CD spectra for functionalized iridium nanoparticles.

Circular dichroism (CD) measurements of the Ir functionalized nanoparticles were carried out (Figure 4). Solutions of l- and d-cysteine produced CD spectra that were almost mirrored images of each other with peaks at 200 nm (Figure 4a), owing to their chiral centers, which makes them optically active. The slight differences in peak intensity can be attributed to small discrepancies in the concentration of the solutions. On the other hand, MPA and cysteamine solutions display no peaks as they are not optically active and no chiral centers are present in these molecules. Like l- and d-cysteine solutions, measurements of IrNp@LCy and IrNp@DCy resulted in similar spectra with peaks red-shifted by 10 nm, appearing at 210 nm (Figure 4b). The presence of albeit weak peaks over the absorbance range of the nanoparticles is also an indication of induced chirality into the nanoparticles.^55−58^ As expected, IrNp, IrNp@CyA, and IrNp@MPA show no optical activity.

### Effect of Chiral Molecular Functionalization on the OER Activity

**Figure 5:**
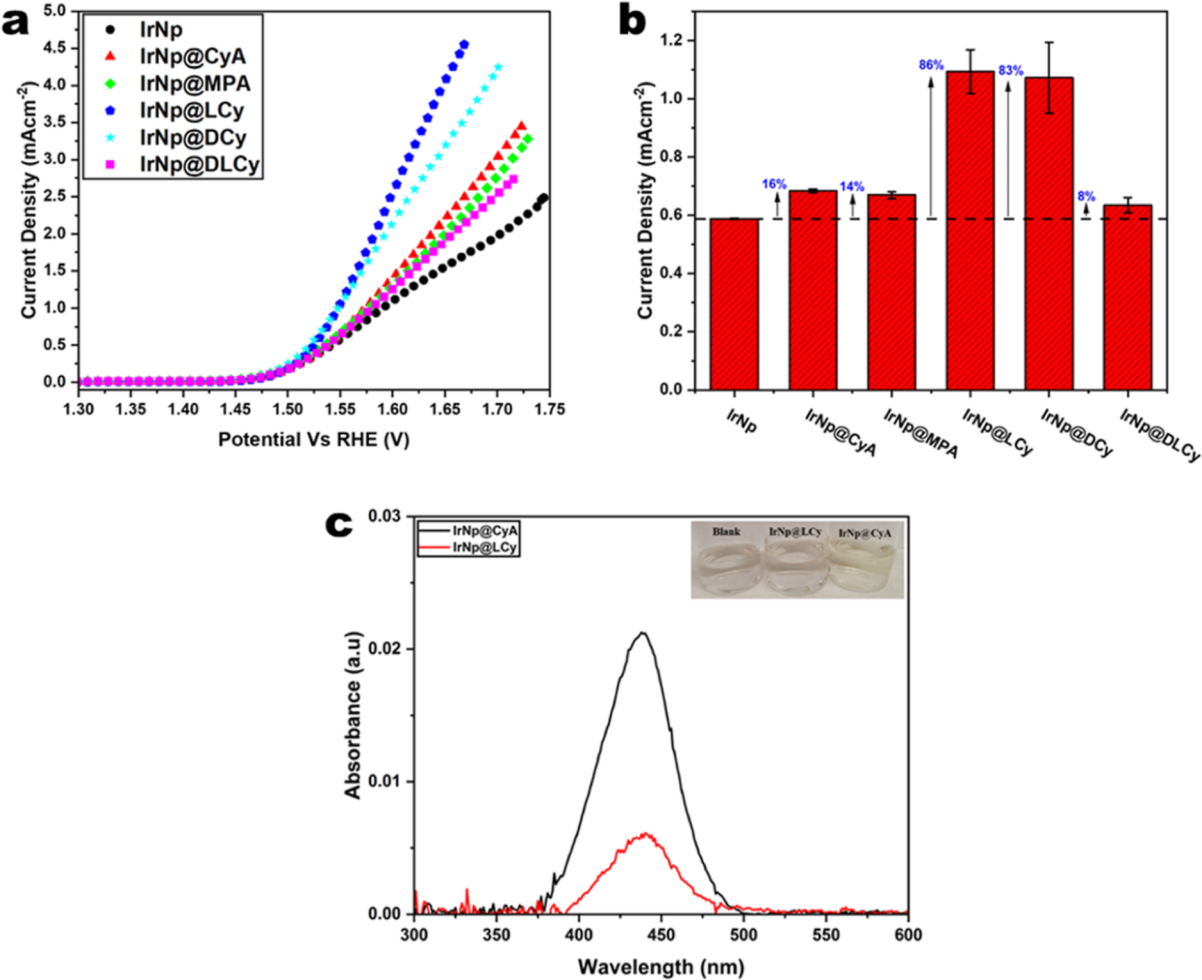
Functionalized iridium nanoparticles activity toward OER. (a) OER polarization curves, (b) ECSA normalized current density at 1.55 V vs RHE, and (c) H_2_O_2_ quantification.

Having confirmed the successful functionalization of the Ir nanoparticles with both chiral and achiral organic molecules, their electrochemical activities toward the OER were probed to assess the effects of the chirality in the electrochemical activity of the Ir nanoparticles. Clear enhancements were observed for all functionalized Ir nanoparticles with respect to the nonfunctionalized ones. Figure 5a shows the linear sweep voltammetry polarization curves for all sample activities toward the OER. At a potential of 1.55 V vs RHE, the percentage increases relative to the nonfunctionalized Ir nanoparticles were 16, 14, 86, and 83% for IrNp@CyA, IrNp@MPA, IrNp@LCy, and IrNp@DCy, respectively (Figure 5b).

The enhancement in activity for chiral functionalized nanoparticles (IrNp@LCy and IrNp@DCy) is attributed predominantly to the CISS effect.^32^ It was suggested that the electron spin selectivity at a catalyst surface can strongly influence the binding and electron transfer between the catalyst and the reactive species. There are studies that demonstrate this efficient electron transfer in electron spin selectivity.^38^ Also, an electron traversing a chiral electric field requires its spin and momentum to be flipped to be backscattered, and as such the probability of this happening during OER on a chiral catalyst is reduced.^25,31,38,59^

There is also an observable enhancement in the level of the OER activity from achiral ligand functionalization (IrNp@CyA and IrNp@MPA) relative to that of unfunctionalized IrNp. This is arguably due to the surface passivation regulating the electronic structure of the catalysts leading to a more efficient OER.^46^ There is an argument to be made that a percentage of the enhanced OER activity of chiral functionalized samples is also due to this effect. Given the similarity in the molecular structures of achiral and chiral ligands used in this study, it however does not justify the much larger discrepancies between the activities, going from achiral to chiral functionalized Ir nanoparticles and, thus, this larger enhancement is attributed to the CISS effect gained from chiral functionalization.

Both achiral molecules were selected to independently evaluate the effect of the amino acid (CyA) and carboxylic acid (MPA) functional groups on the catalytic performance of the Nanoparticles (NPs). While differing in carbon backbone length, the molecules were selected to keep the functional group position consistent with respect to the chiral analogues LCy and DCy, respectively. This was designed as such to demonstrate that the enhanced activity was dominated by the CISS effect and not by the molecular functionalities, both of which are present in the chiral molecules. Both types of achiral samples displayed similar magnitudes of enhanced activities, which infers neither their functional groups nor the relative positions of those groups to the thiol linkers have any significant impact on their ability to affect the current density enhancement. With this deduction, it is safe to assume that the major enhancements of the chiral samples are justifiable due predominantly to the chirality of those ligands as a whole and not because of their functional groups or the relative positions of those groups.

A sample of iridium nanoparticles functionalized with a racemic mixture of d and l cysteine (IrNp@DLCy) was also prepared to substantiate these claims. Functionalization in this manner renders the nanoparticles racemic like the other achiral samples, and thus as is observed in Figure 5a, they demonstrated similar activities to the other achiral samples (IrNp@CyA and IrNp@MPA). The relative percentage enhancement relative to bare IrNP was 8% (Figure 5b).

### Tafel Analysis

**Figure 6:**
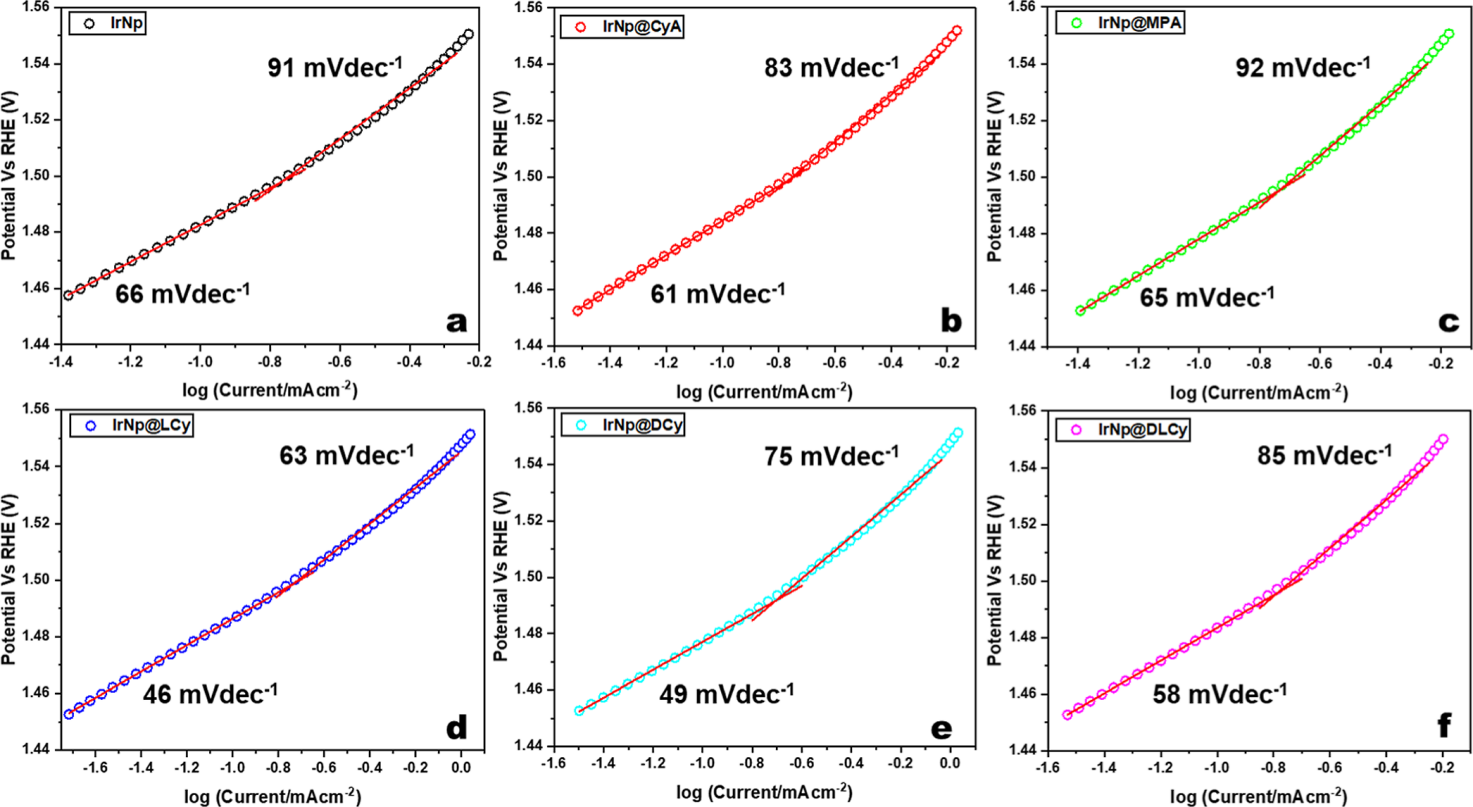
Tafel plots for (a) IrNp, (b) IrNp@CyA, (c) IrNp@MPA, (d) IrNp@LCy, (e) IrNp@DCy, and (f) IrNp@DLCy.

The corresponding Tafel plots are shown in Figure 6. Two distinct linear trends for the lower and higher overpotential (LOP and HOP) regions are observable for all of the samples. The slopes in the LOP regions are 66, 61, 65, 58, 46, and 49 mV dec^–1^ for IrNp, IrNp@CyA, IrNp@MPA, IrNp@LCy, IrNp@DCy, and IrNp@DLCy, respectively. The lower slopes for the chiral nanoparticles, IrNp@LCy and IrNp@DCy, indicate faster kinetics for the OER among the series of materials.

Moving to great overpotentials, the slopes increase to 91, 83, 92, 63, 75, and 85 mV dec^–1^, respectively. These increases are most likely due to an increase in mass transport resistance attributed to oxygen bubble formation on the electrode surface, hindering OH^–^ adsorption and electron transport in the double layer.^60^ Nonetheless, other effects such as change of active sites due to double-layer reconstruction and adsorption of reaction intermediates are all viable possibilities.

For this Tafel analysis, the mechanistic path for OER proposed by Krasil’shchikov’s path was considered.^60−63^ This is laid out step by step in eqs 1 through 4. Where the M symbol stands for a surface site and thus, for example, MOH is a surface-bound OH species. Matching the Tafel slopes obtained from the LOP regions to the slopes of the respective Krasil’shchikov’s path, it is notable that the rate-determining step (RDS) for IrNp@LCy and IrNp@DCy aligns best to the mechanistic step outlined in eq 3, whereas for IrNp, IrNp@CyA, and IrNp@MPA aligns best to the mechanistic step outlined in eq 2.
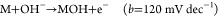
1
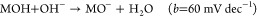
2
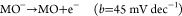
3
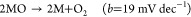
4

Based on the reaction outlined in eq 3, the reaction step that controls the overall rate of the OER on IrNp@LCy and IrNp@DCy is an electron transfer step to generate MO intermediate species. Assuming this is done spin selectively, it creates the environment for selective triplet O_2_ formation.^43^ As explained above, electron transfer at spin-selective surfaces can occur more efficiently. Correlating this with the fact that the RDS of the chiral samples is an electron transfer step supports the notion that electron spin selectivity is responsible for the observed OER enhancement o. On the other hand, the reaction that limits the rate of the OER on IrNp, IrNp@CyA, IrNp@MPA, and IrNp@DLCy is a chemical step which is kinetically less efficient than the RDS of the chiral samples. It also lacks control over the formation of oxygen intermediates, such as the chiral samples. This proposed mechanism is by no means conclusive; however, the correlations between the RDS, the proposed mechanism, and the electrochemical activity are interesting and warrant more exploration in the future. Nonetheless, it is sufficient to say that the reaction step that more efficiently leads to the formation of oxygen would also more efficiently not lead to the formation of other side products and supports the proposed theory and assumptions. This supports the above discussion related to the enhancement of the activity observed.

### Colorimetric Determination of H_2_O_2_

Probing the formation of H_2_O_2_ during the OER is a good approach to test the chiral molecular functionalization effect on product selectivity, as H_2_O_2_ is expected to be reduced at a spin-polarized surface.^38,41^ H_2_O_2_ formation is determined indirectly via a colorimetric method described in the literature and outlined in the Experimental Section.^41,43^ Titrating the reaction electrolyte with *o*-tolidine as a redox indicator causes the clear solution to become yellow as a result of o-tolidine undergoing complete oxidation by H_2_O_2_. The absorbance of the solution is measured, giving a characteristic absorbance band at 436 nm, whose intensity is used to quantify/indicate the amount of H_2_O_2_ present.

The electrolytes obtained from the electrochemical experiments with both IrNp@CyA and IrNp@LCy show this characteristic peak at 436 nm (Figure 5c) indicating the production of H_2_O_2_ during water oxidation. However, chiral functionalized IrNp@LCy produced significantly less hydrogen peroxide than achiral IrNp@CyA, evident from the lower absorbance. This is because the formation is spin-forbidden at a spin-polarized surface due to OH intermediates having parallel spin alignment. This finding is consistent with the CISS effect in water-splitting as previously reported.^22,31,41^

## Conclusions

In summary, iridium nanoparticles were successfully prepared via a simple thermal reduction method and subsequently functionalized with organic molecules of different chirality. Functionalizing iridium nanoparticles with both achiral and chiral ligands has been demonstrated to improve their electrocatalytic activity with respect to nonfunctionalized iridium nanoparticles, with the chiral molecular functionalized samples averaging an 85% enhancement and achiral functionalized nanoparticles averaging a 13% enhancement. The results suggest the chirality imparted on the catalysts via functionalization is predominantly accountable for the enhancement in OER activity observed, which is supported by the reduced H_2_O_2_ production of chiral iridium nanoparticle samples in comparison to the achiral iridium nanoparticles. Tafel plot analysis of these samples was able to determine and distinguish the RDS for all samples, highlighting the more efficient path of the chiral samples and justifying why they outperform the other nonchiral samples.

The implication of these results is the ability to impart chirality into single nanoparticle electrocatalysts to improve their activities toward the OER and suppress the formation of parasitic species via chiral spin selectivity.

## Experimental Section

### Chemicals

l-Cysteine (Merck), d-cysteine (Merck), 1,2-propanediol (Acros Organics), iridium chloride hydrate (IrCl_3_·*x*H_2_O, Merck), cysteamine (Merck), 3-mercaptopropionic acid (Merck), 37% hydrochloric acid (Fischer Scientific), ethanol (99.5% purity, Honeywell), acetone (99.5% purity, Acros Organics), and isopropyl alcohol (99.9% purity IPA, VWR).

### Nanoparticle Synthesis and Functionalization

To synthesize iridium nanoparticles, 10 mg of iridium chloride hydrate was added to 10 mL of 1,2-propanediol in a 50 mL round-bottom flask. The mixture was homogenized with the aid of bath sonication, resulting in a clear yellow solution. This round-bottom flask containing the precursor solution of iridium chloride was attached to a reflux column and heated under reflux conditions for 30 min by way of an oil bath. Within a few minutes of heating, the solution quickly changed from the clear yellow precursor solution color to a dark brown color that remained as such for the remainder of the reaction. After 30 min, the flask was removed from the oil bath and allowed to cool to room temperature. The resulting iridium nanoparticle solution was then subsequently sonicated and collected from the round-bottom flask for further functionalization or washed to collect unfunctionalized iridium nanoparticles (washing is described in the subsection below).

Starting with the unfunctionalized nanoparticles synthesized as outlined above, the functionalized nanoparticles were prepared. To 5 mL aliquots of the as-prepared unfunctionalized iridium nanoparticle solution placed in glass vials, 5 mL volumes of 50 mM solutions of the desired ligands respectively (l- and d-cysteine, cysteamine, and 3-mercaptopropionic acid) were added. The mixtures were left to continuously stir overnight on a magnetic stirrer using stirring bars. Following this period of continuous stirring, the respective mixtures were collected in flacon tubes, where equal volumes (10 mL) of 2 M HCl aqueous solutions were added to wash the nanoparticles. The solutions were sonicated for a few minutes before being centrifuged at 14,000 rpm for 10 min to remove all solvent. The supernatants were collected and redispersed in a small volume of ethanol before they were dried under vacuum. Unfunctionalized iridium nanoparticles are washed and dried in the same manner. Note that to produce the four types of functionalized iridium nanoparticles, multiple batches of iridium nanoparticles were synthesized. Prepared samples were labeled; accordingly, IrNp, IrNP@CyA, IrNp@MPA, IrNp@LCy, and IrNp@DCy for unfunctionalized iridium nanoparticles, cysteamine, mercaptopropionic acid, l-cysteine, and d-cysteine functionalized iridium nanoparticles, respectively.

### Characterizations

TEM images were recorded on a JOEL JEM-F200 Cold-FEG S/TEM operating at an acceleration voltage of 200 kV. Nanoparticles dispersed in DI water were diluted with ethanol before being drop-cast on holey carbon/Cu TEM grids. XRD spectra were recorded in a Panalytical Xpert Pro diffractometer with a Cu Kα radiation source (1.5418 Å). XPS measurements were carried out with a Thermo Scientific NEXSA XPS system equipped with an Al Kα X-ray source. UV–vis absorbance measurements were carried out on a PerkinElmer LAMBDa 35 V-Vis spectrometer. CD measurements were carried out with a Chirascan CD spectrometer from Applied Photophysics.

### Electrochemistry

The electrocatalytic performance of all functionalized and nonfunctionalized Ir nanoparticles toward the OER was examined in a three-electrode system with a Metrohm Autolab PGSTAT302N potentiostat, where a Hg/HgO reference electrode was used as the reference electrode and a platinum coil was used as the counter electrode. Ink solutions were prepared by dispersing 0.5 mg of powdered nanoparticles in a solvent mixture composed of ethanol (0.2 mL), water (0.78 mL), and Nafion (0.02 mL). Ink mixtures in glass vials were submerged in an ice bath and sonicated with an ultrasonication probe for 30 min. Three microliters of the prepared inks were drop-cast and left to air-dry at a rotation speed of 800 rpm on a glassy carbon RDE from Metrohm (geometric area of electrode 0.071 cm^2^).

Linear sweep voltammograms were collected in 0.1 M KOH. Electrochemical scans were done between the potential ranges of 0.1 and 1.8 V vs RHE. The electrical impedance spectrometry (EIS) measurements were conducted as reported by Watzele et al.^64^ The spectra were measured at the potential of 1.47 V vs RHE close to the “onset” potential of the OER, where reaction intermediates cover all active sites of the catalysts without interruptions from O_2_ bubble formation. Measurements were made in the frequency range of 100 kHz to 0.1 Hz with a probing signal amplitude of 10 mV. Electrocatalytic surface area (ECSA) estimated from EIS measurements and the specific absorbance capacitance of 72 μF cm^–2^ reported by Watzele et al.^64^ were used to normalize current densities.

### H_2_O_2_ Determination

Chronoamperometry was run on samples for 30 min in 0.25 M aqueous Na_2_CO_3_ electrolyte. To a 3 mL volume of the used electrolyte, 1 mL of o-tolidine solution prepared according to the Elmms–Hauser method was added and allowed to react for 30 min before the solution was measured in a PerkinElmer LAMBDA 35 spectrometer.

## Supporting Information Available

It contains further characterization of the materials prepared, specifically: optical images from the synthesis process of Ir nanoparticles; transmission electron microscopy images of Ir nanoparticles and particle size distribution data; XRD patterns for Ir nanoparticles and table with (*hkl*) peak positions for each Ir sample; UV–vis absorbance spectra and EIS spectra for Ir samples; and schematic representation of the *O*-tolidine oxidation process (PDF)
