# Supplementary material for: Electron Spin Selective Iridium Electrocatalysts for the Oxygen Evolution Reaction
Source: ACS Mater Au. 2023 Nov 29;4(2):204–13. doi: 10.1021/acsmaterialsau.3c00084 (PMC10941284; doi:10.1021/acsmaterialsau.3c00084)
Supplement: Supplementary file 1 — mg3c00084_si_001.pdf [file mg3c00084_si_001.pdf]

Electron Spin Selective Iridium Electrocatalysts for Oxygen Evolution Reaction

Carlos J. Mingoes [a], Bob C. Schroeder [b], Ana B. Jorge Sobrido [a]**

[a] School of Engineering and Materials Science, Queen Mary University of London*

[b] Chemistry Department, University College London

Supporting Information: Images depicting nanoparticle synthesis, TEM images of nanoparticles, XRD spectra and peak positions break down for all samples. UV-Vis absorbance spectra and electrical impedance (EIS) spectra for all samples. Schematic for O-tolidine oxidation.

List of contents

Figures

Figure S 1 . Images of (a) IrCl3.xH2O precursor solution, and (b)IrNps. 2

Figure S 2. TEM images and the corresponding core size histograms for (a, b, c) IrNp@MPA and (d, e, f) IrNp@LCy. 2

Figure S 3. XRD spectra for IrNp, IrNp@CyA, IrNp@MPA, IrNp@LCy and IrNp@DCy nanoparticles. 3

Figure S 4. Absorbance spectrum for a) ligands, b) functionalised iridium nanoparticles. 4

Figure S 5. Fitted impedance spectrums for all functionalized IrNp. Inset is the equivalent electric circuit where R_{Ω} is the uncompensated resistance, CPE_{dl} is the double layer impedance, R_{ct} is the charge transfer resistance, R_a is the adsorption 5

Table

Table S 1. XRD peak position assignments for functionalised IrNps. 4

Scheme

Scheme S 1. *O*-tolidine oxidation..6

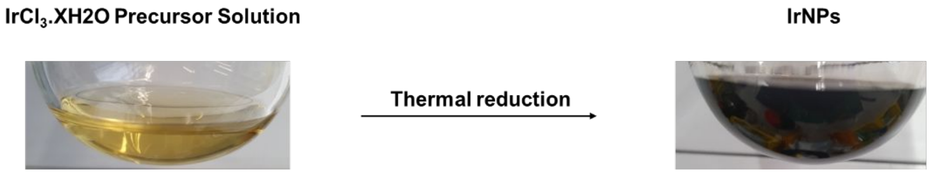

Figure S 1 . Images of (a) IrCl3.xH2O precursor solution, and (b)IrNps.

Images

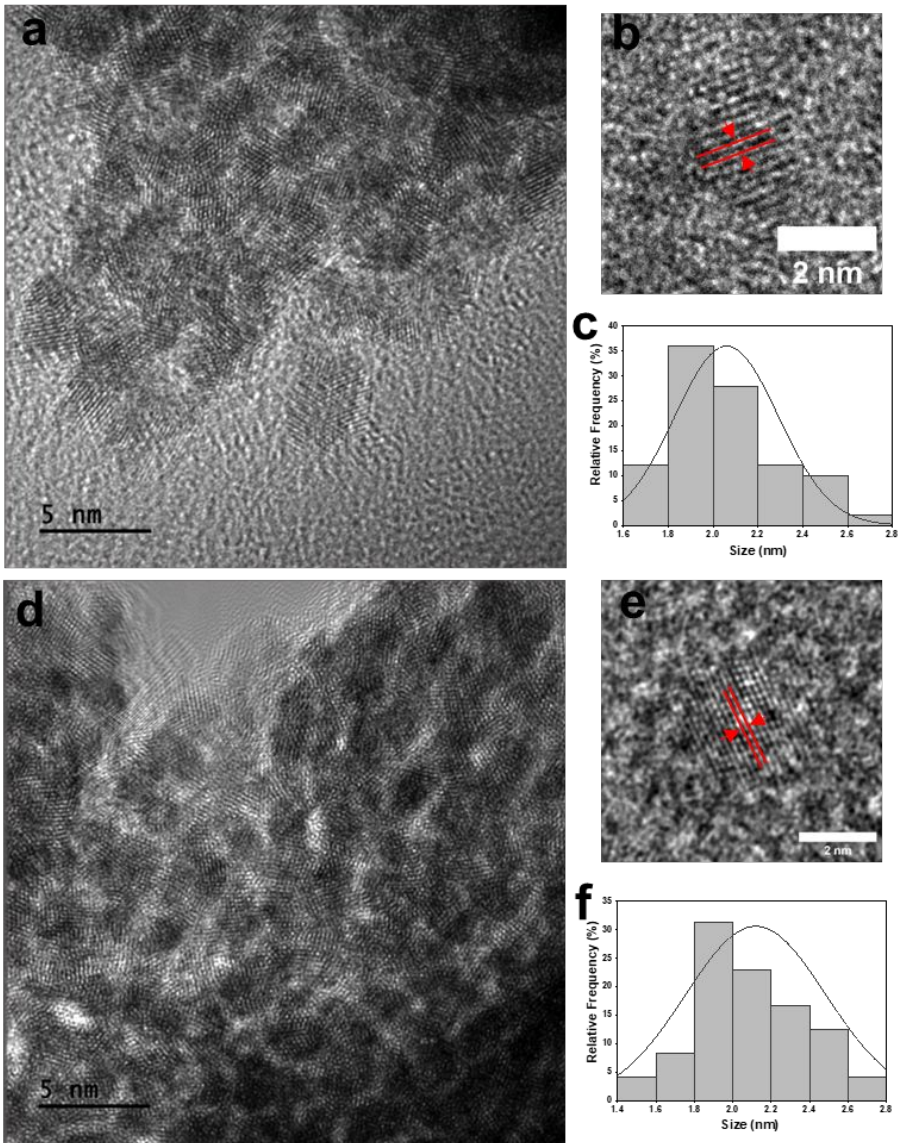

Figure S 2. TEM images and the corresponding core size histograms for (a, b, c) IrNp@MPA and (d, e, f) IrNp@LCy.

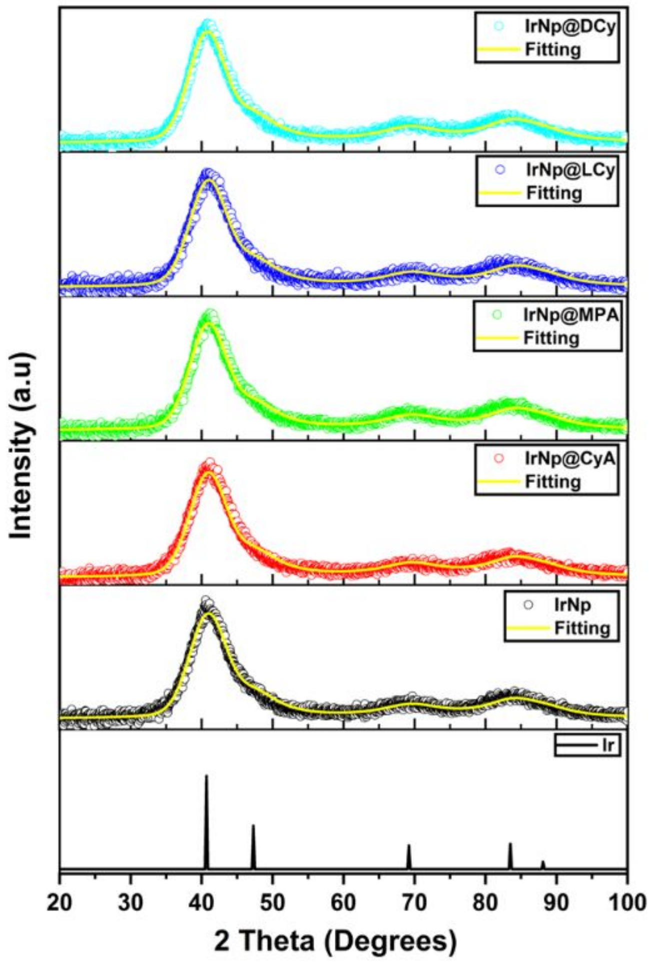

Figure S 3. XRD spectra for IrNp, IrNp@CyA, IrNp@MPA, IrNp@LCy and IrNp@DCy nanoparticles.

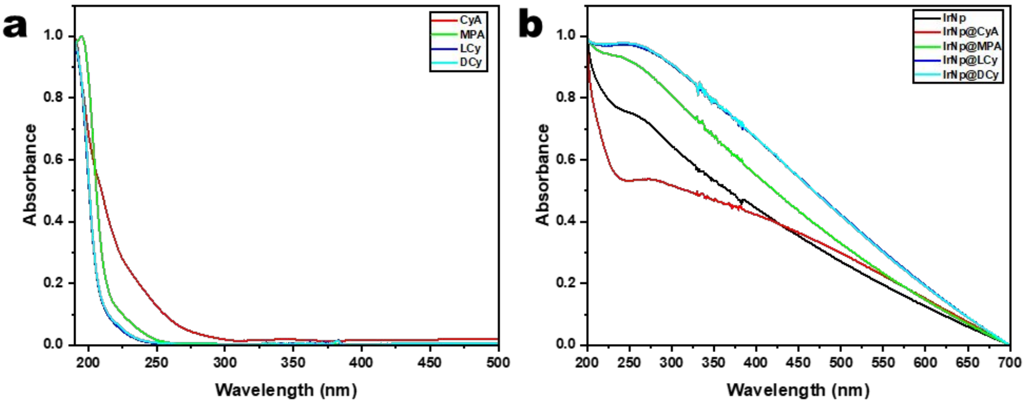

Figure S 4. Absorbance spectrum for a) ligands, b) functionalised iridium nanoparticles.

Table S 1. XRD peak position assignments for functionalised IrNps.

h k l	Ir (standard)	IrNp	IrNp@CyA	IrNp@MPA	IrNp@LCy	IrNp@DCy
	Peak Position (2θ)					
**111**	40.7	40.84	40.88	40.86	4.88	40.71
**200**	47.35	47.52	47.56	47.54	47.56	47.36
**220**	69.21	69.47	69.53	69.51	69.54	69.22
**311**	83.51	83.85	83.9	83.89	83.94	83.52
**222**	88.14	88.51	88.6	88.56	88.61	88.16

ECSA Determination

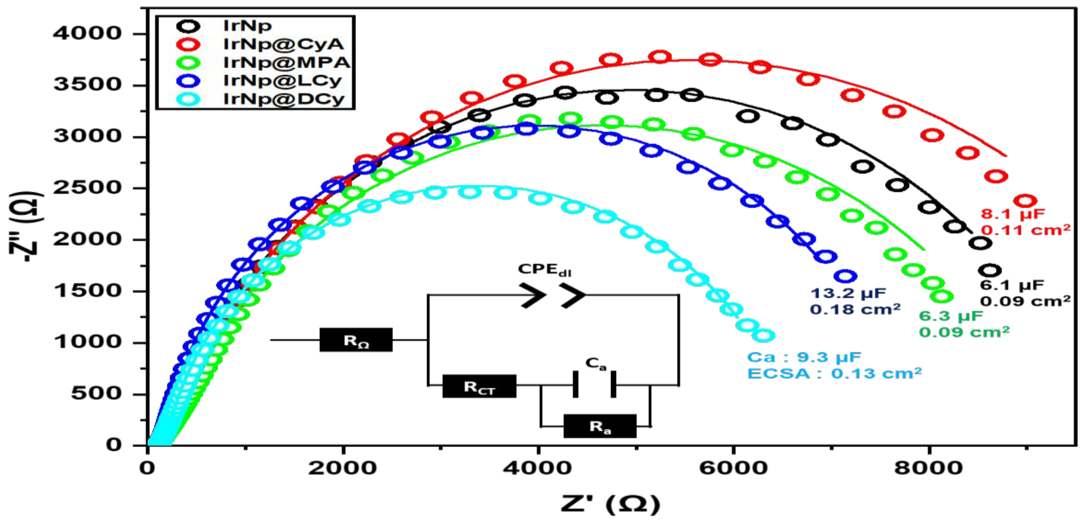

Figure S 5. Fitted impedance spectrums for all functionalized IrNp. Inset is the equivalent electric circuit where R_{Ω} is the uncompensated resistance, CPE_{dl} is the double layer impedance, R_{ct} is the charge transfer resistance, R_a is the adsorption

ECSA for samples were determined by an electrical impedance spectroscopy (EIS) method. EIS measurements around the onset potential were done to determine the adsorption capacitance (C_a) which was used to calculate the ECSA of all measured samples. Examples of the data obtained from these measurements and fittings can be observed in figure S5. The spectrums were fitted with the equivalent electric circuit shown in the inset, where R_{Ω} is the uncompensated ohmic resistance, CPE_{dl} is the double layer capacitance represented as a constant phase element, R_{ct} is the charge transfer resistance, and C_a and R_a are the adsorption capacitance and adsorption resistance respectively.

C_a and R_a are influenced by the catalyst's interaction with surface intermediate species, where C_a is proportional to the concentration of surface reactive intermediates and to the ECSA. The displayed C_a and ECSA obtained from the fittings were used to produce the normalised LSVs. The differences in R_a are due to the slight differences in activities recorded around the onset potential of 1.47 V vs RHE used for all samples. R_a which is associated with the rate of formation of surface intermediates decreases with potential as intermediates becomes easier to form, thus the sample demonstrating the greater activity at the same potential translates to a more efficient surface intermediates formation and lower R_a.

It should be noted that even though there are slight differences in the activities across samples for the potential chosen, due to the overall activity of all samples being very low in this region

(as a result of being in the onset potential region for all samples), the estimation of ECSA across samples is not compromised.

H_2O_2 Determination

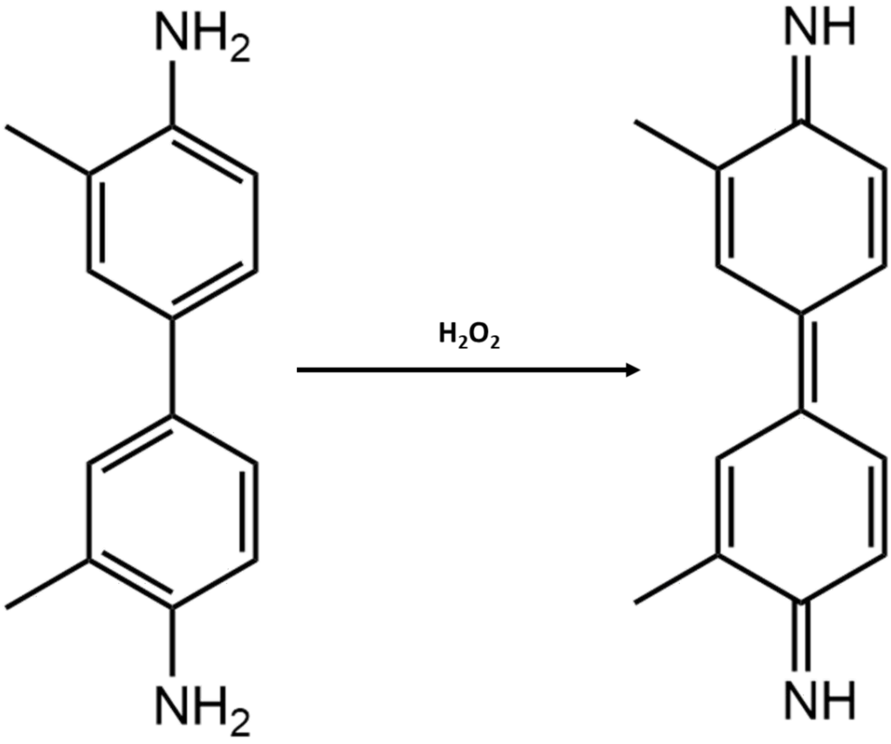

Scheme S 1. *O*-tolidine oxidation
